# Are the natural sciences ready for truth, healing, and reconciliation with Indigenous peoples in Canada? Exploring ‘settler readiness’ at a world-class freshwater research station

**DOI:** 10.1007/s13412-020-00601-0

**Published:** 2020-04-07

**Authors:** Elissa Bozhkov, Chad Walker, Vanessa McCourt, Heather Castleden

**Affiliations:** 1 School of Environmental Studies, Queen’s University, Biosciences Complex, Room 3134, Kingston, Ontario K7L 3N6 Canada; 2 Health, Environments and Communities Research Lab, Department of Geography and Planning, Queen’s University, Mac-Corry Building, Room E318, Kingston, K7L 3N6 Canada; 3 Department of Geography, University of Exeter, Amory Building, Rennes Drive, Exeter, UK

**Keywords:** Truth and reconciliation, Water science, Indigenous knowledge, Indigenous-settler relations, Environmental management, Two-Eyed Seeing

## Abstract

The Experimental Lakes Area in Northwestern Ontario, Canada, is a globally prominent freshwater research facility, conducting impactful whole-of-lake experiments on so-called ‘pristine’ lakes and watersheds. These lakes are located in traditional Anishinaabe (Indigenous) territory and the home of 28 Treaty #3 Nations, something rarely acknowledged until now. Indeed, Indigenous peoples in the area have historically been excluded from the research facility’s governance and research. Shortly after it changed hands in 2014—from the federal government to the not-for-profit International Institute of Sustainable Development (IISD)—the Truth and Reconciliation Commission (TRC) of Canada released its Calls to Action to all Canadians. The newly named International Institute of Sustainable Development-Experimental Lakes Area (IISD-ELA) began to respond with a number of initiatives aimed to develop relationships with local Indigenous peoples and communities. In this paper, from the perspectives of IISD-ELA staff members, we share findings from an exploratory study into the relationships beginning to develop between IISD-ELA and Treaty #3 Nations. We used semi-structured interviews (*n* = 10) to identify how staff perceived their initial efforts and contextualize those with the current literature on meaningfully engagement in reconciliation. Our analysis highlights perceived barriers, including time, resources, and funding constraints, as well as an acknowledged lack of cultural awareness and sensitivity training. Participants also recognized the need to engage Indigenous knowledge holders and embrace their ways of knowing at the research station. While the study is small in scale, as an international leader in freshwater science, transparency in the IISD-ELA’s journey in reconciliation has the potential to inform, influence, and ‘unsettle’ settler-colonial scientists, field stations, and institutions across the country and beyond.

## Introduction

‘Nibi (water) is needed for all life to sustain itself, and we [Anishinabek] are nibi’ Ogamauh annag qwe miinwa Waasaunda qwe Chiblow (2019), p. 11.

**Fig. 1:**
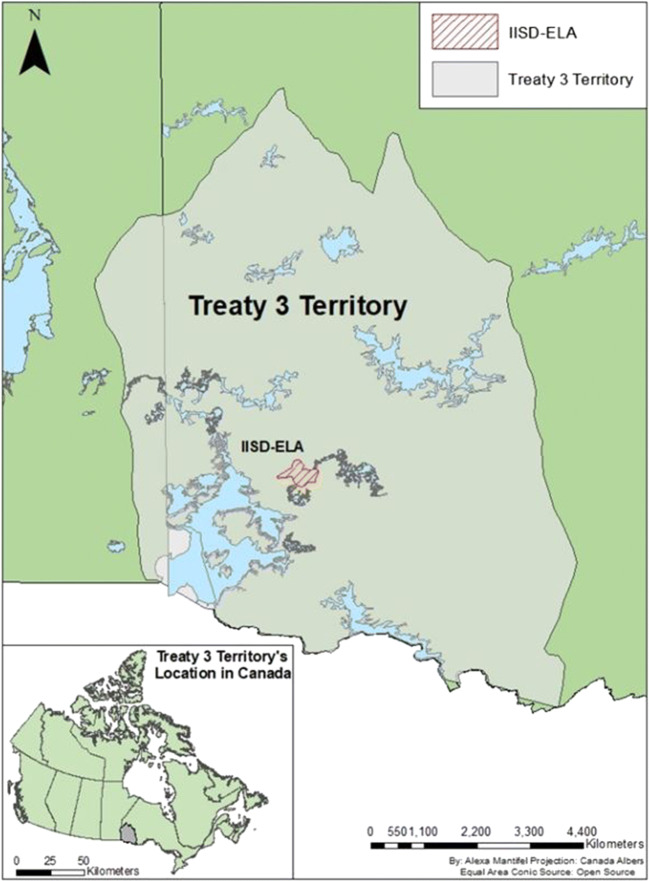
Map of Treaty #3 Territory and the IISD-ELA, overlapping the provinces of Manitoba and Ontario, Canada (© Alexa Mantifel)

Located on Treaty #3 Territory^1^ [see Fig. 1] in the present-day Kenora district of Northwestern Ontario, the Experimental Lakes Area (ELA) is home to 58 freshwater lakes and is an internationally renowned freshwater research station (International Institute for Sustainable Development - Experimental Lakes Area (IISD- ELA) 2017a, c). Through manipulation of ELA lakes and watersheds, aquatic researchers trained in Western science traditions are able to conduct real-world experiments (Blanchfield et al. 2009). The ELA’s research activities have spanned over 50 years and have been described in over one thousand peer-reviewed scientific publications (IISD-ELA 2017a). Such research contributes to a long-term understanding of freshwater ecosystems and influences environmental policies around the world.

Since its inception in 1968, research at the ELA had been directed by Fisheries and Oceans Canada (DFO), a federal department within the Government of Canada, and thus was severely inhibited by governmental restrictions (Orihel and Schindler 2014). Despite its world-class standing, in 2012, the then Conservative government of former Canadian Prime Minister Stephen Harper gave notice to withdraw funding to the ELA citing the need to save on operating costs. In an effort to save the ELA and sustain the tradition of basic scientific discovery, a spirited campaign was initiated by scientists from around the world (Hoag 2013). Success followed in April 2014 when the International Institute of Sustainable Development (IISD)^2^ signed a number of agreements with the Government of Ontario and the Government of Canada ensuring the long-term operation of the ELA. Xenopoulos and Frost (2015) wrote that “the ‘new’ ELA is better positioned to promote and build capacity for informing freshwater and ecosystem policy around the world” (p. 87). Part of this optimism came from the IISD-ELA’s desire to engage more with the public—including local Indigenous communities—through their research programmes (IISD-ELA 2017c; Xenopoulos and Frost 2015).

It is within this context that we report on our investigation into what ‘meaningful engagement’ with the original Indigenous land stewards of the area looks like for the staff of the IISD-ELA. This research is particularly important in the post-Truth and Reconciliation era in Canada, wherein there was a national apology and Commission into the cultural genocide of Indigenous peoples through ‘Indian Residential Schools’ and where Canada recently adopted the United Nations Declaration on the Rights of Indigenous Peoples (UNDRIP) without qualification (UN General Assembly 2007). Given the change in ownership, as well as the Truth and Reconciliation Commission’s (TRC) final report and 94 Calls to Action arising from its 5-year inquiry, the time is ripe for this kind of exploratory research that will help us increase our understanding of Indigenous-settler relations and institutional ‘readiness’ for truths about settler-colonialism, and—hopefully—consequent healing of relationships, and authentic reconciliation efforts in the natural sciences.

## Background

By acquiring the ELA, the IISD made a commitment to preserve its scientific foundation, while doing so in the context of *participatory* and *inclusive* research (Xenopoulos and Frost 2015). This contrasts with most natural scientific pursuits, which are conducted as investigator-driven experiments. Historically, (non-Indigenous) researchers would direct and carry out research *on* people, including Indigenous people, rather than *with* them (see Castleden et al. 2012; Maclean & TBYBI, 2015). This trend has been seen across the natural, health, and social sciences, where research is said to be disconnected from locality and ‘the practical problems of policy’ (Merton 1949, p.161; see also Dieleman et al. 2019; Palmer 2012). For many Indigenous peoples, the term ‘research’ has become a ‘dirty word’ (Smith 2013, p. 1). This is due to the reality that research on or about them has a long history of exploitation and misrepresentation—causing more harm than good in many circumstances (Smith 2013). Not surprisingly, there has been a fair degree of Indigenous scepticism about Western approaches to all kinds of science, especially as Indigenous ontologies, epistemologies, and methodologies have been ignored, neglected, and dismissed (Kermoal and Altamirano-Jiménez 2016). Mi’kmaw scholar, Marie Battiste (2002) cautions that Western-centred views of knowledge production should be considered a form of imperialism that purposefully erases other types of knowledge.

In contrast to Western science, Indigenous knowledge (sometimes called IK, traditional knowledge, or Indigenous sciences as in Johnson et al. 2016) is ‘acquired through experience and observations or from spiritual teachings, and handed down from generation to generation’ (p. 350, Ford et al. 2016), with proven value ‘in the field’, and in classrooms of environmental studies and sciences (see Moore 2012; Rich 2012; Kimmerer 2013, 2012). Debassige (2013) reminds us that ‘Indigenous peoples are the original researchers of these territories’ (p. 16) and yet despite their value, Indigenous knowledge holders are often seen and treated as out of place in academic institutions where it is ‘easy’ to reproduce colonial relationships (Baijius and Patrick 2019; Johnson et al. 2016). Settler-scholars with interest in working with Indigenous peoples must recognize the many challenges (see Zanotti and Palomino-Schalscha 2016) and ‘learn to see our own privilege…our deep colonizing’ (Johnson et al. 2016; p. 3). In the context of environmental decision-making, von der Porten et al. (2016) have warned that ‘using IK’ in a superficial or secondary sense does not address the root of problem—one born out of colonial structures, not scientific and technical pursuits, as advertised (Curran 2019). Limited participation of Indigenous peoples in such decision-making may only serve the interests of those already in power by creating an image of legitimacy (Curran 2019; Schilling-Vacaflor 2017).

Research that is conducted in the silos of Western scientific disciplines is further problematic in light of specific calls for environmental research to be more collaborative and inclusive of Indigenous peoples and their knowledge systems (Berkes 2009; Big-Canoe and Richmond 2014; Ward-Fear et al. 2019). In Canada, all university-based scientists, in order to hold Tri-Agency funding, must comply with the Tri-Council Policy Statement (TCPS2) on Research Involving Human Participants, including Chapter 9, which addresses research involving the First Nations, Inuit, and Métis Peoples of Canada (Government of Canada 2018). This was established to create an ethical space for dialogue on common interests and points of difference between researchers and Indigenous communities engaged in research (CIHR 2018). The challenge here is that many natural scientists and engineers have not been trained in a culture of ethical space that includes responsibilities to the land (and water, and all living—and non-living—entities) and thus fail to consider how the TCPS2 has application to their research (Ermine 2007; Castellano 2014; Kershaw et al. 2014).

Most relevant to this paper, Indigenous knowledge is said to have particular value in the study of water because it helps to change how people think about and relate to the resource (Stefanelli et al. 2017). In the Canadian context, Western science alone has proven to be ineffective at addressing water-related challenges within Indigenous communities (Sanderson et al. 2015; White et al. 2012). Across the world, Kimmerer (2013) has claimed that Western science is ‘asleep at the wheel’ as rivers are contaminated and water systems are on the verge of collapse. A collection of papers from a 2019 special issue in *Water* (see Wilson et al. 2019a) reveal some important insights regarding the tensions between Indigenous and colonial water governance. One of the big ‘takeaways’ from these articles is that Indigenous understandings of water is of a living entity—which conflicts with Western-orientated views of water as a resource for exploitation (Wilson et al. 2019a; see also McGregor 2014; Yates et al. 2017). Especially when set within colonial water governance practices which are biased toward technical and scientific approaches (Cavazos Cohn et al. 2019), such a conflict results in the water crises all too common in Indigenous communities in Canada (and beyond). Accordingly, such ‘water problems’ should be accurately framed as ‘political problems’ or ‘problems of governance’ (Baijius and Patrick 2019; Taylor et al. 2019; Wilson et al. 2019b).

As noted above, another call for change comes from the 2015 Final Report of the TRC. The report urges every corner of Canada to fully adopt and implement the United Nations Declaration on the Rights of Indigenous Peoples (UNDRIP). Because there are no specific Calls to Action for the non-profit research sector, for this paper, we draw inspiration from the TRC Call to Action #65, which calls on the Federal government (via SSHRC) to develop a national research programme to advance our collaborative understandings of reconciliation, and Call to Action #92, which calls on the corporate sector in Canada to adopt UNDRIP as a reconciliation framework and to apply it to core operational activities involving Indigenous peoples, lands, and resources. UNDRIP is also important to our understanding of water (as in the present study) because it has been used as the ‘normative backbone’ of Indigenous water rights and justice (as in Taylor et al. 2019; Robison et al. 2018). Doing so may help to reframe the way we think about and govern water in terms of Indigenous (i.e. Anishinbek as in Chiblow 2019) relationships. Lastly, we turn to Call to Action #53(ii), demanding evaluation on reconciliation efforts across all of Canada (Truth and Reconciliation Commission of Canada (TRC) 2015).

Given the need for the following: (i) reconciliation between the science community and Indigenous peoples and (ii) healing relationships with the land, this study explores the institutional readiness of a world-class science station, the IISD-ELA, to engage with local Indigenous communities in Treaty #3 territory in the spirit of truth, healing, and reconciliation. As outlined above, there is plenty of recent research on the tensions between Indigenous and Western scientific *water governance*—including on how working toward healthy and clean water futures can help to rebuild fractured relationships between Indigenous peoples (Taylor et al. 2019; Poelina et al. 2019)—but much less study on the extent to which those tensions arise in *water science*. This research aims to inform not only initiatives at the IISD-ELA, but environmental management and science research at other institutions (i.e. ENGOs as in Gordon 2018) where there is genuine interest in engaging Indigenous ontologies, epistemologies, and methodologies.

### Research context

Following their takeover of the ELA, one of the first steps in the IISD’s Indigenous engagement initiative involved hosting a ‘Fall Feast’, beginning in 2015 and now held annually. A chance meeting in 2016 between the corresponding author and the Executive Director of the IISD-ELA resulted in an invitation to attend the third annual Fall Feast (September 2017), and to explore the possibility of carrying out an ‘institutional readiness for reconciliation’ research project. The first and corresponding authors of this paper attended the gathering, engaged in participant observation, and began developing relationships for the research. Approximately 40 people attended, with roughly 60% associated with the ELA and 40% from surrounding Indigenous (Anishinaabe) nations of Migisi Sahgaigan (Eagle Lake), Naotkamegwanning (Whitefish Bay), and Shoal Lake 40. The Feast took place out of doors, by the water, a sacred fire was kept by a traditional fire-keeper for the duration of the event, and an Indigenous drum group sang. A conversation circle about the ELA ensued, and the event wrapped up with a chef-prepared gourmet banquet.

After the Fall Feast, we received endorsement from the IISD-ELA Director and Deputy Director—and later informed consent from individual staff members—to move ahead with this collaborative study. It is important to note that the research findings in this paper represent the first part of a larger programme of research concerning the IISD-ELA’s efforts in reconciling relations with Indigenous peoples in Treaty #3. We determined it was appropriate to first focus on the perspectives of the IISD-ELA staff in order to gain insight on their understanding of reconciliation and current engagement efforts thus far. There were also important ethical elements of our decision. Developing new research relationships with Indigenous communities takes time to build trust and respect (Castleden et al. 2012) to the point where settlers/outsiders (Dwyer and Buckle 2009)—even those with a history of working with other Indigenous communities—may be welcome to begin a study. The next step of this research has been involving exploratory conversations with Treaty #3 representatives to determine their level of interest in engaging in research about the reconciliation journey at IISD-ELA.

## Methods

Ten staff members (out of a total of twenty invited) volunteered to participate in this study vis-à-vis semi-structured, open-ended, qualitative interviews; the protocol received clearance by the General Research Ethics Board of Queen’s University. This method was chosen because interviews are best suited to investigate the diversity of meaning, opinion, and experiences that we sought (Hay 2016). They also had practical value given the relatively small number of staff members (20) at the IISD-ELA and for the opportunity to speak freely without concern of judgement from peers. The inductive nature of the interviews further allowed for adaptability to address new issues that were brought to the discussion by participants (Becker et al. 2002). The open-ended interviewing strategy allowed each participant to explore the topic into areas of their interest in relation to the overarching research question (Weller et al. 2018). Participants were cautioned that given the small sample to recruit from and that we were identifying the organization in publication, there was a real risk to being identified (or misidentified); those who agreed to participate did so with informed understanding of the risks and benefits of the study and gave their consent.

Our research team developed an interview guide that consisted of 18 questions organized around three major areas of interest: (i) the participant’s job description and work history, (ii) personal engagement or knowledge of local (or any) Indigenous cultures, and (iii) opinions on current and future opportunities for engagement with Indigenous people in Treaty #3. The first area provided a window on the type of job/obligations each staff member held and how things may have changed since IISD assumed responsibility for the ELA. The next two areas delved further into the individual participant’s current experiences and ideas for the future as they relate to engagement, relationship building, and reconciliation with Indigenous peoples. All interviews were conducted in Fall 2017 by the first author. They were audio recorded, and transcribed verbatim.

In the first round of data analysis, a conventional content analysis approach was used to code categories derived from the interviews. This approach uses initial code keywords and involved counting and comparisons of such words throughout the interview texts (Hsieh and Shannon 2005). From there, two rounds of thematic analysis, where content was sorted into meaningful clusters based on linking ideas, took place (Patton 2002). This type of analysis avoids the use of preconceived categories and instead allows for the creation of newly formed insights to emerge from the data, a common approach in qualitative inquiry (Kondracki and Wellman 2002).

## Findings

Our findings are organized around three themes: (1) how IISD-ELA staff perceive changes that have occurred since the ELA left governmental control; (2) barriers that are still in place preventing meaningful engagement and partnership with local Indigenous peoples; and (3) participant-identified opportunities for overcoming such barriers. A summary of these results (plus wider recommendations) can be found in Table 1 below. While direct quotes from interviews are used to showcase themes, participants were given numbers (P#1–10) to seek protection of their identities. Field notes from participant observations, as well as larger discussions around effective Indigenous-settler engagement, are woven into our findings. The purpose here is not to be critical of a particular individual or group of individuals, but rather to point out the challenges that exist, which are problematic everywhere, in terms of moving toward reconciling relationships and knowledge systems.

**Table 1:** Summary of findings and recommendations

Themes	Sub-themes
1.Changes since IISD ‘took over’	• ‘Opening doors’ to local First Nations communities
2. Existing barriers to meaningful engagement	• Lack of time • Lack of resources • The need for better historical and cultural understandings
3. Opportunities to overcome barriers	• Incorporating local First Nations’ history, culture, and language BUT, making sure not to ‘native wash’ • More direct involvement in research activities (i.e. through citizen science, community-based monitoring) • Hiring [local] Indigenous peoples in science roles
Wider recommendations	
• Further research to look at meaningful engagement with local Treaty #3 First Nations • Application of Etuaptmumk and utilizing Indigenous knowledge in research • Incorporation of more Indigenous content (history, cultural understandings) in Canadian education • Action on all 94 Calls to Action of the Truth and Reconciliation Committee • Application of the UNDRIP principle of Free, Prior, and Informed Consent in Environmental Science	

### ‘Opening doors’ at the ELA

Many participants we spoke to described how their role—and indeed the overall mandate of the ELA—has changed dramatically since the IISD took over. For example, P#10 describes a facility that was mostly closed to the public, with a bad reputation as a ‘top secret’ research site.

In the early years, when the facility first started under the federal government…, it was remote, restricted access. Didn’t do a whole lot of engagement of any kind with the public, Indigenous or non-Indigenous. …We were getting a bad reputation with the local public of having this top secret, remote research facility- creating three eyed fish, that kind of stuff.

Another participant (P#4) told us how things had changed for the better over the past few years. They describe how the previous facility ran under ‘layers of bureaucracy’ which made it difficult to engage with or in local communities.

With respect to First Nations, it’s [now] much easier to engage with local community. We used to have many layers of bureaucracy in the government, very afraid of what was said would be interpreted as official government policy, very difficult to engage in communities as part of the government. Now… [the IISD-ELA] is very actively interested with communicating what we’re finding, with interacting with communities. A huge change that we’re all still adjusting to.

Their reference to the ‘huge change’ suggests some positive initiatives are now in place, but that they are still in an ‘adjustment’ period in terms of making sense of this new moral/ethical responsibility for meaningful engagement with local First Nations. Thus, the IISD-ELA may be in a kind of transition period—a time when colonial structures and Western science are being rightfully challenged by Indigenous science and knowledge holders (Battiste 2002). One participant (P#6) talked about the theme of positive change using a long-term, organizational perspective. They, and other participants, described the emergence of outreach at the ELA as being novel and exploratory and that reaching these associated goals would take some time.

If we started this outreach decades ago it would be much further along than it is today. The fact that it’s a new programme, it’s always hard to get things going.

This participant recognized that in an ideal world, the IISD-ELA, regardless of it being—or perhaps because it was—a former government-controlled lab, would have already had well-established, meaningful relationships with Treaty #3 communities. Under new direction, it was acknowledged that outreach staff now has a lot of work to do. Again, this indicates the research centre is in a transition zone—not where they want to be, but in a ‘better place’ than they were just a few years ago.

### Beginning the work of meaningful engagement

While changes were starting to occur at the IISD-ELA, such initiatives were admittedly in their infancy. Beginning such work is never easy and many participants spoke about the barriers they saw as preventing such goals of community-engaged Indigenous research. Below, we share participants’ perspectives of the three main barriers.

#### Time: tensions between desirability and feasibility

Many participants noted a lack of time as being a major barrier to deepening further engagement with Indigenous communities. When asked: ‘On a scale of 1-10, what is your interest in visiting nearby Indigenous communities to share what your work is about at the IISD-ELA?’, P#7 indicated that although they had interest and could do more to engage, they identified sufficient time as an issue.

My interest is eight or nine. Unfortunately, my ability would probably be a two just because I don’t have time. My job is pretty intensive — that’s why I propose they come to us…. All of the scientists want to help, we’d love to go teach them. We’re already so busy, we have limited time. I think everyone would love to.

On the one hand, it is encouraging to see an interest in engaging in reconciliation efforts. On the other hand, it is concerning that there is a perception that ‘help’ is wanted or needed. Essentially, three issues are at play here. First, there is no recognition about how busy Indigenous communities may be and yet the onus is on them to come to the IISD-ELA. Second, there is a lack of awareness and acknowledgement that Indigenous knowledge-holders are already considered environmental experts in their own right. And third, that Indigenous peoples want to or need to be ‘taught’. That is, there is a perception that Indigenous peoples are the ones who ‘require’ education and there is a lack of awareness or ‘readiness’ to be ‘the learner’ in Indigenous spaces, taught by Indigenous peoples.

When asked about the impact of interacting with visitors on a participant’s workload, P#2 describes such visits as ‘interfering’ with the research schedule.

I’d say the big activities that require ‘all hands on deck’, it interferes with the daily work schedule and we’re all really busy on the research end. We all think it’s important, but also striking a balance — I got to carve out another 2 hours to give my spiel on the… program. That gets taxing. I don’t think the outreach isn’t important. But you don’t need the actual research scientist doing that.

Meanwhile, P#9 notes that community outreach was not the reason they were hired at the IISD-ELA.

We as science staff are really happy to talk to people when they are on site and visit communities to do presentations — that definitely happens, but we definitely need people that can arrange things and work on the outreach as their main focus. Whereas for the scientists, our science has to be our focus, that’s the reason we’re here, and that the ELA exists, so that balance really has to be struck.

Here, we see that engagement with visitors, including the Treaty #3 Nations, was perceived as a ‘welcome inconvenience’. It is seen as a disruption to the science taking place, not an opportunity for mutual co-learning. The underlying message is that reconciling relations between the IISD-ELA and the original land and water stewards should be kept within the purview of outreach staff, and that this was not a priority for Western-trained scientists to engage with, or at least spend time to facilitate such communication with Indigenous scientists (i.e. traditional ecological knowledge holders). This kind of discourse emerged within what we observed as silos of responsibility (for the ELA and for reconciliation) between the different staff roles. Scientists, although having an interest in engagement, indicated that their top priority was research. P#9 for example, expresses interest in sharing their scientific work to visitors; however, they do not express a perspective that seeks to identify opportunities to collaboratively work with Indigenous knowledge holders. This participant, and others expressing similar sentiments, suggests that the dominant view at IISD-ELA is that Western science should continue to remain the main form of inquiry.

At the same time, participants involved in management and operations indicated that they had more time to focus on community and Indigenous engagement. P#1 states that:

Three quarters of my time is spent on education outreach. I also build relationships with First Nations communities in Treaty #3, which has included meetings with First Nations communities trying to understand their concerns in terms of freshwater in the region and trying to see if there’s ways that we can do work in collaboration to address concerns.

On face value, this type of engagement is an important process, though what we do not hear about or yet see in practice is how or to what extent the conversations result in action and change. Outside of the annual Fall Feast—which only occurs for one afternoon a year—it was not clear what other action had resulted from the engagement or how Indigenous peoples are contributing their insights into the scientific work at the IISD-ELA. Here, the management and operations staff state that they spend three quarters of their time on outreach, yet the science staff report a lack of time for community and Indigenous engagement, which reiterates the disconnect present between the commonly accepted Western science practices at ELA and that of traditional IK. The science staff’s interest in going to ‘teach’ Indigenous communities and ‘give presentations’ on their individual work demonstrate an unintentional yet prevalent perspective, which supports the colonialist structure of contemporary freshwater science. That IISD-ELA as a scientific field station finds it difficult to make space for IK to be a leading form of knowledge or even to contribute to the scientific research conducted there reinforces the notion that Western science is ‘the way’ and wholly separate from that of Indigenous peoples’ systems of science.

#### Resources: Never enough

Another key barrier mentioned by participants was a lack of resources for both scientific research programmes and engagement/outreach programmes. References to limited resources were identified across three interrelated categories: (i) having the staff available to carry out engagement activities, (ii) having the physical infrastructure in place, and (iii) having the financial resources to operationalize activities. Some, like P#7, mentioned the small number of total staff at the IISD-ELA (*n* = 20) meant they were often unable to do all the things they would like to.

We do not have the capacity or resources in house. We have a pretty streamlined staff as it is, and our staff is already working really hard to get done what we have to get done. We’re kind of over-committed. So we’d have to hire more people to make [engagement] happen.

Sentiment like this was common and suggests that the small team is doing all they can—at least given their current responsibilities and areas of expertise—to make engagement ‘happen’. And while opinions differed regarding how such staffing issues could be addressed, some participants, like P#4 and P#10, shared optimistic views of future engagement. They noted that because overall interest was clear, such barriers were not really that substantial and could easily be overcome through more resources—especially money.

Money is a big thing. I think the [other] barriers are not that great. I think there’s a lot of interest on the part of the IISD. (P#4)

Maybe I’m naive, but I think within our group there aren’t a whole lot of barriers. I think this is something that everyone’s on board with. With me it’s capacity in terms of infrastructure. (P#10)

Again, the staff we spoke with most often pointed their proverbial fingers toward factors outside of their control when speaking about the lack of meaningful engagement. Meanwhile, P#10 expanded on the challenges of securing funding and how more financial support would be needed to develop the physical infrastructure on IISD-ELA’s site to accommodate larger-scale engagement activities. For them, this included culturally sensitive and suitable infrastructure for large community gatherings, meetings, or sharing circles.

As a non-governmental organization we don’t have the money for this kind of infrastructure. So, we have to go out and find sources...originally this site was designed just to house our own internal research staff, the site was never designed to accommodate the outreach activities, so we didn’t put in infrastructure that could hold 50-60 people at one time.

The point this participant is making is that even if there were high levels of institutional will and human resources, the physical space may be a limitation for engaging in reconciliation activities at the IISD-ELA. Others we spoke with, like P#8, went further with regard to a lack of resources and noted that the federal and provincial government should do more in terms of providing monetary support.

I think in terms of money, if the federal government were to be serious about reconciliation, there would be money available for the type of things we’re doing, so it wouldn’t be so precarious.

Several participants acknowledge a lack of money to be a major component in both beginning community engagement efforts and sustaining them in the long run. The transition from an institution run by the government departmental budget to a not-for-profit organization means that the ELA has to find funding from different outside sources, which participants state place limitations on engagement efforts. With unstable funding in this neoliberal climate, the impacts experienced by non-governmental organizations like the IISD-ELA will make it difficult to keep pace with current reconciliation efforts, many of which only scratch the surface, let alone dwell deeply into what truth, healing, and reconciliation can and should be now and into the future.

#### The need for cultural awareness training

The third area that participants consistently brought up was a lack of knowledge about the socio-cultural history of the land on which they work, and Indigenous peoples more generally. Almost all participants (like P#9 and P#10) were forthright in acknowledging their time growing up—including their university training—which was void of teaching about the lived realities of Indigenous peoples and our colonial history.

I don’t think [my Indigenous history knowledge] is as broad as it should be. I know about the different groups throughout Canada…having done work in different areas. I know general information about the residential schools, followed that a little bit in the news. I’ve never done any Native studies courses or through school that looked at history or environmental relations. So, most of the knowledge has been gained personally through the news, reading, and talking to people. No formal education. (P#9)

I would say in the earlier years, very little [knowledge of Indigenous history]. Personally, growing up, the environment I was in, I had very little exposure to First Nation communities. I didn’t acquire that exposure on a regular process until I was out at the ELA area. (P#10)

Participants candidly point to the knowledge gap evident throughout their education background, including a combinative lack of formal and informal exposure to Indigenous peoples’ histories and knowledge systems. When asked about the Indigenous history of the land, most staff members were unable to go beyond identifying the land as Treaty #3 territory. Yet, like P#6, many of those interviewed recognized their limited knowledge and expressed an interest to address this.

I’m learning every day, more and more about some of the components about what it means to be on Treaty land and how that came to be, and how severely colonialism has impacted these communities, and how unaware we are in Canada as a society. I’m sure that [non-Indigenous] people that live on Treaty #3 land have no idea they’re on Treaty land. I feel like I still have a ton to learn but there’s definitely staff here at ELA that know less than I do.

The self-admitted ignorance we heard is certainly common in many quarters of Canadian society; regardless, it is troublesome given the staff work and live on Treaty #3 land and are thus in a contemporary Treaty relationship though none expressed familiarity with the provisions of that Treaty. Yet, through these interviews, the desire for improvement seemed genuine. Apart from simply admitting their lack of knowledge, some participants went further and questioned the process of how to incorporate such cultural awareness to achieve effective and meaningful reconciliation. Notably, the differences between Western approaches to science and Indigenous knowledge systems were brought up. P#4 spoke about how Western-trained scientists are not provided with training in other systems of knowledge and are unfamiliar with how to go about working with communities—Indigenous or otherwise.

It’s partly a culture thing, primarily the ELA has come from a place of doing hard core Western science. Data collection, writing proposals, etc. We don’t have any history of working with communities so I know I am very comfortable working in the Western sciences — it’s what I’ve learned and grown up doing in my professional life. I can move in that world. When we go out of that world, my comfort level is a little less and my suspicion is that it’s like that for most people.

This feeling of unpreparedness was common amongst participants. P#3 notes that while they see the value of Indigenous knowledge and involvement in scientific research covering the oil sands and mining, they had difficulty envisioning how to draw on such knowledge with respect to the science undertaken at the IISD-ELA.

To be honest I’ve struggled with this question at ELA. I understand and appreciate the incorporation of traditional knowledge for environmental assessments for mines, but it’s much more complicated to envision it at ELA specifically. Maybe I just lack imagination.

Indeed, this comment represented the perspectives of many IISD-ELA participants; it highlights the unease or perception of being unable to think outside the box, especially stepping outside of their traditionally Western science training. On the one hand, the fact that P#3 mentioned that they have ‘struggled with [the] question’ of Indigenous involvement may indicate they are willing to look for ways to do so in the future. On the other hand, it may indicate that they are not investing (or willing to invest) in developing their imagination to envision how Indigenous knowledge can play any role.

When asked about their past and current engagement experiences at IISD-ELA, participants’ responses were varied. One example of how these differed can be seen through how they spoke about Indigenous cultures and perspectives during the annual Fall Feast. Some participants detailed numerous occasions where they participated in meaningful engagement, while others felt there was limited to no engagement. P#5 felt like both IISD-ELA and the Treaty #3 peoples who attended gained a lot from the experience.

The one group brought some of their drummers out, some of the folks were wearing traditional dress, they danced, they sang, they drummed. For me, I’m passionate about music so I thought it to be very entertaining to get insight into that part of the culture… It’s an opportunity for us to look through a different lens at the same place, but from a different perspective. I’d like to believe both parties gain a deeper sense of the value or potential of this landscape and its resources by understanding how someone else is utilizing or managing or monitoring them.

While this participant speaks to how the event allowed them to ‘see’ the ELA through a different lens, the fact that they describe the cultural/ceremonial activities as ‘entertaining’, although it does indicate a level of appreciation, it also suggests they may have experienced the event without fully understanding the weight that ceremonial roles hold for such gatherings of Indigenous peoples. Because most of the land in Canada has been stolen or treatied under duress, it may be difficult for non-Indigenous peoples to grasp the fraught terrain they are on. This ignorance may be born out the inadequate national narrative or the education about Indigenous-settler history (see Godlewska et al. 2010).

Others, like P#9 were more outwardly critical about the effectiveness and meaningfulness of the Fall Feast. They shared concerns that perhaps there was little to no substantive benefit from the event, for the IISD-ELA staff as well as those attending from nearby Indigenous communities, beyond tokenistic expressions.

I’m a little concerned that initially it was regarded as a good thing - I think there are good intentions on everybody’s part. After the first couple times it was like ‘nice to meet you’. I’m worried as the years pass we just say hi to each other and go our separate ways and that’s not enough. We’re in need of an active, more constructive engagement where each side, especially the First Nations side has something they can take home. And I’m not sure that’s happening. From my perspective it’s not from a lack of trying from IISD, at times it’s hard to know how to provide meaningful assistance or something we can actually do to make a difference, to make people feel like this is worthwhile. There’s a feel-good aspect but I’m not sure that’s enough.

Participants we spoke with noted that in the coming years, more would be needed to deepen and improve these relationships. The level of critical self-assessment in participant responses show that some IISD-ELA staff can and do identify limitations to their current engagement efforts and reveals a need to develop more active and effective collaboration moving forward. Although a good starting point for relationship building, at the time of our study, the question remained, for the staff of the ELA, about how to actually move the Fall Feast and similar engagements beyond ‘saying hello’; to move from superficial encounters to real, meaningful engagement based on the Articles of UNDRIP. That IISD-ELA is inviting the neighbouring First Nations to come and join *them* on ‘their turf’ risks putting the Western ways of being and knowing at the fore and the drumming as entertainment.

### Opportunities for reconciliation at the ELA

Given there is much to be improved upon, participants spoke about solutions to overcoming the barriers mentioned above and more long-term opportunities to improving engagement at the IISD-ELA. Most participants noted that the IISD-ELA has the potential and the ability to do more; however, they appreciated that the means to achieve it and the logistics required to do so would need to be addressed.

#### Making tangible change at the ELA

P#5 spoke of recognizing infrastructure barriers, but also how they were easily remedied through the engagement and incorporation of Indigenous cultural values. For instance, with regard to the ELA’s decision to build a sacred fire pit, P#8 learned about what kind of design elements to put into place that would be respectful to local First Nations’ beliefs and spirituality and acted on it.

[The interaction with our neighbours has] highlighted to me possible changes to our infrastructure on site to support some of this work — i.e. a recommendation from one of the earlier Fall Feasts suggested that we need to build a sacred fire pit. The one we were using wasn’t culturally appropriate, so we identified a suitable area, had a sign erected and built one that was more in line with the cultural values of the community.

Examples like these were described as fairly simple fixes, though participants pointed out that assumptions should not be made about infrastructure being culturally suitable without prior, clear communication with the neighbouring Indigenous communities. To see people like P#8 admit they did not know the ‘right answer’ when it came to things like the fire pit is encouraging. In another example of a staff member trying to address cultural awareness and language at the IISD-ELA, P#6 told of an outreach project they were working on:

Right now we’re working on trying to translate one of our infographic videos to Ojibway, so we’re working with an Indigenous community member from near Fort Francis. We’re looking to have an Indigenous Ojibway language specialist come and work with our staff about cultural awareness and language.

This outreach project is a step in the right direction as it recognizes and shows a level of respect for Indigenous knowledge—by inviting an Indigenous expert to teach ELA staff, it demonstrates that IK can inform and guide learning for science staff.

#### Changing the status quo

While the two initiatives above suggest some progress in terms of relationship building and greater understanding of the local Indigenous culture, there were concerns expressed during one interview that certain types of engagement efforts may not always be genuine. P#1 thoughtfully leaves open the possibility that their idea of conduct might be seen not only as insincere by Indigenous peoples, but could also provide a false sense of accomplishment:

What I’m wondering is there some sort of ceremony that we should be doing as part of our experiments or at the beginning I don’t know if that would help, would show that we are incorporating traditional values into our work. But I would never want to be seen as ‘native-washing’ to be crass, in the same way like ‘green-washing’ so there’s an interesting thing to think about there, it’s a difficult balance… I don’t know.

This staff member’s choice of words in using ‘show’ and ‘seen’ indicates a worry in engaging on superficial terms and perhaps how that may reflect negatively on the organization as a whole. The participant’s concern on being perceived as ‘native-washing’ again further reinforces the unease that Western scientists have in stepping outside of their traditional training—hesitancy both in unknowing how to proceed and also in fear of being ‘insensitive’—which often inhibits people from engaging with communities unfamiliar-to-them at all.

Participants further suggested establishing a community-based monitoring programme as a meaningful mechanism to bring Indigenous knowledge that comes from trans-generational stewardship of the land into relationship with the interests and skills of Western-trained scientists. Participants spoke of the benefits that such a programme would provide for both the IISD-ELA and the First Nations involved in terms of approaching environmental problems. P#7 brought up the importance of making sure that monitoring programmes are fundamentally community-driven in leadership and implementation.

These communities need data of what’s in the environment for not only their own knowledge, to know what’s going on, but so when they go to go sit at the table with people that make the decisions they are armed with knowledge .... They expressed some distrust of when university academics come, they do the monitoring, they may not share it and then they leave.

However, P#1 questioned how to go about that logistically:

How do you incorporate traditional knowledge into our oil spill project when there’s not necessarily traditional knowledge on oil spills? Someone recently pointed out to me at a Treaty 3 meeting that there may not be traditional knowledge but there may be community knowledge from communities that have experienced oil spills.

These participants reinforce the need to ensure a community feels comfortable in its relationships with researchers prior to and while research is being conducted, as previously Indigenous communities have had damaging experiences with Western researchers. Although these comments acknowledge an awareness for Indigenous knowledge to be included in scientific studies, its incorporation is still uncertain. Moreover, there is a historicization of traditional knowledge in this remark, as if such knowledge is only of the way things were, not the way things are. Indigenous knowledge holders bring the teachings of the past and apply that knowledge to contemporary issues, and so it would be a missed opportunity not to engage Indigenous knowledge holders in oil spill research or any other contemporary issue (e.g. microplastics, pesticides, and so on).

P#8 shared that one of the Anishinaabe Nations under Treaty #3 had approached the IISD-ELA with interest in doing a citizen science^3^ initiative. The staff expressed excitement to partner on this initiative.

We thought the citizen science would be about certain water quality parameters but we heard from the community was that what they were interested in was mercury and fish and wild rice. So, we’re working with the community in developing citizen science about mercury levels in fish and wild rice.

This observation and resulting action highlight just how important it is to engage communities at the start, before even determining what research topic should be of focus. After further conversation with the community, P#8 learned that their research interests, along with fellow scientists, differed noticeably from those of the community. Recognizing these differences before formulating the research question—and indeed, before problem identification—has allowed the IISD-ELA scientists to work with respect in their collaboration with the Treaty #3 Nation. One tangible way to help promote these processes was seen in the interview with P#10, who stated that visits and discussions with local populations created the biggest impact. They describe listening to community concerns and problems—and acting upon them—helps scientists perform meaningful research.

I would say the biggest impact is what comes from those individuals on site. Where do they see the future concerns for themselves as citizens? Both Indigenous and non-Indigenous. Environmental threats to their well-being, their communities’ well-being. What are the next issues we should be researching? I think that’s a big part of moving forward at IISD-ELA, making sure that we’re practical and relevant with our research.

On the whole, participants recognized the interconnectedness of environmental management with community involvement and development. They acknowledged the importance of community stakeholders in collaborative roles to not only help inform, but also produce research that is both useful and addresses local community concerns.

#### Indigenous staff at the ELA

Finally, when speaking to potential solutions to address a lack of time and capacity amongst current staff, participants suggested hiring local, Indigenous knowledge holders could be a valuable next step. P#7 noted that it would provide an employment opportunity in a low-employment region and would also allow the researchers to have more time to focus on their own scientific work.

We’d get the work done that we need to get done. It wouldn’t take too much more resources for us because these are positions we’re hiring for anyways. It would provide skills for them to take back to their communities and conduct it in their own communities.

Hiring Indigenous knowledge holders as staff could provide benefits for both community development and help to inform and guide science research objectives to be locally relevant. Others like P#1 suggested that in order to address differences in research interests and values, establishing an Indigenous Advisory Body and/or hiring an Indigenous scientist, could effectively bridge communication between the science station and surrounding communities.

We should establish an Advisory Body or a group of Elders that we pay. I think that there would be value for sure. And we would love, even our research team has talked about how it would be great to get to have a First Nations (person) to work as a biologist.

Participants shared their belief that hiring full-time staff whose sole responsibility would be to help move along the ELA’s work with relationship building, facilitating scientific input as needed from communities, and participating in events going on in the nearby region would greatly benefit both the ELA and the Indigenous communities in the territory. While this has been the general response across research (academic) institutions across the country, an intense hiring spree, the climate in some contexts remains chilly; that is, the unsettling work needed to decolonize individuals and institutions needs to happen alongside the creation of space for Indigenous peoples and knowledge systems too.

## Discussion

Given the TRC Calls to Action for all Canadians, including natural and environmental research scientists, to commit to truth, healing, and reconciliation efforts, this research aimed to increase scholarly understanding of staff-perceived initiatives at the IISD-ELA in Treaty #3 territory. Thus, this study is a direct response to the TRC Calls to Action #65, #92, and #53(ii), with the latter calling for the need to ‘to monitor, evaluate, and report on reconciliation progress across all levels and sectors of Canadian society’. In light of our findings, there are a number of important takeaways that may help research and other institutions similarly pursuing Indigenous-settler reconciliation. We see these helping to inform those in the natural sciences in Canada and other colonial states.

One of the main messages through early discussion with staff members of the IISD-ELA was a noticeable difference in levels of engagement from when the Canadian government was overseeing the research station. Participants reported secrecy and isolation surrounding their scientific work. Although research at the IISD-ELA has informed global policy for decades, historically, it has had limited contact with the general public, and especially the local Treaty #3 communities. This aligns with the penchant for natural, health, and social scientists in other realms to do the same, which has resulted in making research a ‘dirty word’ (Smith 2013, p. 1). Now with IISD at the helm of such a highly influential research facility, there has been a push for better communication, community outreach, and education. The participants of this study recognized this change as the first step in a more genuine, community-driven practice of environmental science.

While our results showed that the IISD-ELA has been starting to take on positive initiatives to engage with Indigenous peoples, staff members confessed they were still in a transition period. More can and needs to be done to meaningfully engage local Treaty #3 First Nations in the post-Truth and Reconciliation era. These changes were admittedly difficult, likely made more so by still-present perceptions of Western science as superior to local Indigenous knowledge or sciences (Johnson et al. 2016)—an idea deeply engrained in our common understanding of knowledge production (Battiste 2002). This has been shown to be especially prevalent in the halls of academia, where settlers’ work often reproduces colonial relationships (Baijius and Patrick 2019; Johnson et al. 2016). This is problematic especially in the study of water as IK has proven valuable (Sanderson et al. 2015; Stefanelli et al. 2017; White et al. 2012) and Western water science has been seen as being ‘asleep at the wheel’ (Kimmerer 2013).

The barriers that staff members mentioned during this study included a lack of time, resources, and cultural awareness and understanding. These ideas were not spoken about as ‘excuses’, but rather participants gave important thought to the opportunities that could address such barriers and were often critically analysing them as a way to work toward better long-term relations with local First Nations. That said, the IISD-ELA and others interested in genuine engagement and reconciliation through water science would be well-served by acknowledging the dangers in limited participation or superficial actions. In what one of our participants called ‘native-washing’, these kinds of activities may only make things worse by creating the image of legitimacy (see Curran 2019; Schilling-Vacaflor 2017).

The high interest level of the staff to engage further and learn more about the Indigenous history of the landscape encompassing the IISD-ELA is promising. Though it must be noted that participant reflections on past engagement efforts, including the Fall Feast, varied greatly. Some staff felt the meaningfulness and effectiveness of such endeavours could be improved, whereas others felt like they had already done enough. This begs the question of the following: what is ‘enough’, and who gets to define ‘enough’, in the context of reconciliation in natural and environmental science?

Future work should seek answers to these questions from Indigenous governments, organizations, and peoples themselves in order to embrace a much-needed ‘nothing about us without us’ approach (Ball and Janyst 2008). There is also a clear need for research involving both Indigenous peoples and non-Indigenous water scientists to more fully understand how tensions between the two may be created through conflicting views of water—that is, as a resource or as a living entity. Such questions have been explored in recent research within water governance (Wilson et al. 2019a; see also McGregor 2014; Yates et al. 2017), and water research and management (Castleden et al. 2017a, b, c; Stefanelli et al. 2017). As noted in Castleden and colleagues’ work in water science and governance, both should acknowledge and operationalize the following: (i) UNDRIP as the ‘normative backbone’ of Indigenous water rights (Robison et al. 2018; see also Chiblow 2019) and (ii) that the origin of Indigenous ‘water problems’ is not with communities themselves but lays with colonial policy and governance (Taylor et al. 2019; Wilson et al. 2019b).

One way the IISD-ELA is working with local First Nations communities is through a community-based monitoring (CBM) programme. By switching from water quality to mercury in wild rice, this example highlights the importance of engaging and asking communities (Indigenous and non-Indigenous) to co-define research problems at the start of a project as the values and interests of Western scientists are not always the same as a community. This reinforces the common problems associated with research where there is a disconnect from the problems of locality (Dieleman et al. 2019; Palmer 2012). Even the most well-intentioned researchers can do this, highlighting why place-based knowledge (Indigenous knowledge) is critical in addressing community priorities and formulating research questions. This has been discussed in academic research models—especially CBM and community-based participatory research (CBPR) (see Tobias et al. 2013; Wilson et al. 2018; Arsenault et al. 2019)—and may serve as a model for other NGOs (see Gordon 2018) and other industry professionals.

Involving local, Indigenous communities from the onset of research ideas, designing research objectives and questions with the community undertaking a leadership role is a strategy for studies that aim to decolonize the research landscape (Castleden et al. 2012; Asselin and Basile 2018; David-Chavez 2019). For example, CBM programmes have been said to ‘provide a launch pad for the recognition and inclusion of Indigenous epistemologies and community participation’ (Absolon and Willett 2004, p. 11). Such an approach is critical moving forward, given the long history of research that has been both unethical and exclusive of Indigenous world views. That said, researchers should be aware that doing community-engaged research does require a substantial amount of relationship building (it is *not* just about ‘drinking tea’) before research questions can be explored (see Castleden et al. 2012). Working in such contexts will also require that researchers gain a rich understanding of and use an Indigenous research methodology, not just a Western one. Shawn Wilson (2001) asserts, ‘Indigenous research methodology means talking about relational accountability. As a researcher, you are answering to all your relations when you are doing research’ (p. 177). Doing so may help non-Indigenous academics to merge the traditions and practices of systems they know, with Indigenous knowledges they do not—crafting ‘creative alternatives for conveying spoken knowledge beyond written words’ (Gone 2019, p. 55).

One tangible way the IISD-ELA (and others) may want to think about working within Western scientific and Indigenous knowledge realms is *Etuaptmumk* (or Two-Eyed Seeing)—a Mi’kmaw principle arising from the teachings of Elder Albert Marshall of Eskasoni First Nation (Canada), which may help prevent us from ‘forcing’ IK into Western paradigms of water science (Castleden et al. 2017b. In summary of the idea, Marshall describes that we must learn to:

…see from one eye with the best in our Indigenous ways of knowing, and from the other eye with the best in the Western (or mainstream) ways of knowing…and learn to use both these eyes together, for the benefit of all (p. 2, Marshall and Bartlett 2010; see also Bartlett et al. 2012)

Two-Eyed Seeing is being taken up in many Indigenous contexts, particularly where health research is being undertaken, and also in watershed and environmental management (see Castleden et al. 2017c; Kutz and Tomaselli 2019).

The findings from our study suggest that the lack of cultural awareness and knowledge may hinder the movement of Western-trained scholars out of the traditional scientific paradigm (Baijius and Patrick 2019; Johnson et al. 2016). It is the system they are most familiar with and trusting of, and this stems from a broader structural problem in settler-developed educational systems (Stein 2020). Of all the participants interviewed here, most had a limited working knowledge of Indigenous approaches to science and environmental management. Few had ever taken Native studies courses throughout their education, and especially not within their post-secondary schooling. This kind of collective ignorance (Arrows 2014; Schaefli and Godlewska 2014) contextualizes why most participants struggled to understand what ideas of Indigenous-settler reconciliation, UNDRIP, the TRC Calls to Action meant for them personally or professionally.

Courses in public school curriculum from grades 1 to 12 in Ontario, Canada, contain 1.9% coverage of any Indigenous matters (Godlewska et al. 2010). The omission of Indigenous peoples in our education systems represents a silencing or at least a subliminal white-washing of key issues within society. This allows for problematic narratives to abound, for Indigenous perspectives to be de-valued, and contributes to the neglect of Indigenous ways of knowing in Canada’s Western-based education system (Kapyrka and Dockstator 2012). With much of IISD-ELA’s staff going through this system, it is not surprising for feelings of discomfort or apprehensiveness to arise within Indigenous engagement processes, even where there is genuine interest to do so.

To address the disconnect between Indigenous knowledge systems and Western scientific knowledge systems, participants spoke about the benefits of more cultural awareness and sensitivity training. IISD-ELA staff suggested that to make it happen, there needs to be more hiring, preferably Indigenous peoples or at least with lived experienced in Indigenous knowledge systems. As one participant mentioned, the idea of establishing an Indigenous Advisory Body to facilitate relationship building would benefit both the IISD-ELA and the Treaty #3 communities they share the land with. Doing so would also contribute to Calls to Action #65 and #92. If Canada is to embrace UNDRIP without qualification, then the principles of Free, Prior, and Informed Consent (Schilling-Vacaflor 2017) in science also need to come into play around what happens to the land and waters of Indigenous peoples across the country.

## Conclusion

When engaging in research that involves Indigenous communities, establishing deeply rooted and well-connected relationships is essential for creating trust—especially within the history of settler-colonialism, the current colonial state, and ongoing systemic racism in Canada. The first stage in a genuine and authentic reconciliation endeavour is to understand any pre-determined attitudes, misinformed knowledge held, or institutional barriers that may be present; the science community needs to actively engage in unlearning what they have been taught in schools, through the media, and in their networks. In speaking to IISD-ELA staff, we recognize discourses of improvement alongside a clear room for further growth in terms of the next steps toward meaningful engagement with local Indigenous communities, organizations, and governments. That is, there is a real appetite at IISD-ELA to engage with the Indigenous peoples of Treaty #3.

While this case study is small in terms of its sample size, the IISD-ELA was intentionally studied for its reputation as a world-renowned freshwater science station. That the IISD-ELA was willing to ‘put itself out there’ in a transparent way is commendable. The humility that participants had—a key teaching in many Indigenous societies—to subject themselves to scrutiny and be a ‘demonstration site’ to others about the importance of engaging in reconciliation efforts throughout the environmental sciences, is courageous.

We say ‘courageous’ because just this year, Treaty #3’s Women’s Council and Professor Aimée Craft led the development of a sacred Nibi (Water) Declaration with the Treaty #3 Grand Council and a group of researchers working on the ‘Decolonizing Water project’ (Gray 2018). It was unanimously endorsed at their Chiefs National Assembly (Grand Council Treaty #3 2019). The Declaration includes water law principles and is intended to guide decision-making processes that relate to water and is a Call to Action throughout Treaty #3 to *protect* water. On face value, this could be seen as a tension for IISD-ELA-affiliated scientists who intentionally *pollute* the lakes in the name of science. But IISD-ELA shares many areas of common interest with the Treaty #3 Nations, including environmental impact of resource development, education and youth engagement, and long-term visions of sustainability. And so, when Indigenous and non-Indigenous peoples sit down together, share knowledge through stories over tea (see Castleden et al. 2012), like the IISD-ELA and Treaty #3 are doing, mutual respect, understanding, and maybe even collaboration, can result.

Ultimately, we hope the research outlined here helps to provide some clarity for those wishing to begin or continue to engage in action-oriented and Indigenous community–based research. In the environmental science community and beyond, non-Indigenous Canadians can all do better in ‘imagining’ and acting in reconciliation. We must—particularly in the context of the TRC Calls to Action and our current climate crisis –where the status quo has proven insufficient.

## Footnotes

1 Treaty #3 territory is defined by all lands (in present-day Northwestern Ontario and parts of eastern Manitoba) covered by the October 1873 Treaty 3 signed between the Saulteaux band of the Ojibwa peoples and the Government of Canada (Filice 2019). More information and the full text of the Treaty can be found at: http://gct3.ca/our-nation/our-treaty/

2 ‘The International Institute for Sustainable Development (IISD) is an independent think tank championing sustainable solutions to twenty-first century problems. Their mission is to promote human development and environmental sustainability.’ (IISD-ELA 2017b)

3 Phillips et al. (2012) describe citizen science as a ‘methodology that engages the public in large-scale scientific research while also attempting to achieve social and educational objectives’ (p. 82).
